# The Effect of Systemic Parameters and Baseline Characteristics in Short-Term Response Analysis with Intravitreal Ranibizumab in Treatment-Naive Patients with Neovascular Age-Related Macular Degeneration

**DOI:** 10.3390/pharmaceutics16010105

**Published:** 2024-01-13

**Authors:** Laura García-Quintanilla, Pablo Almuiña-Varela, María José Rodríguez-Cid, María Gil-Martínez, Maximino J. Abraldes, Francisco Gómez-Ulla, Miguel González-Barcia, Cristina Mondelo-García, Ana Estany-Gestal, Francisco J. Otero-Espinar, Maribel Fernández-Rodríguez, Anxo Fernández-Ferreiro

**Affiliations:** 1 Pharmacy Department, University Clinical Hospital of Santiago de Compostela (SERGAS), 15706 Santiago de Compostela, Spain; laura.garcia.quintanilla@sergas.es (L.G.-Q.); miguel.gonzalez.barcia@sergas.es (M.G.-B.); cristina.mondelo.garcia@sergas.es (C.M.-G.); 2 Clinical Pharmacology Group, Health Research Institute of Santiago de Compostela (IDIS), 15706 Santiago de Compostela, Spain; pablo.almuina.varela@sergas.es; 3 Pharmacology, Pharmacy and Pharmaceutical Technology Department, Faculty of Pharmacy, University of Santiago de Compostela (USC), 15782 Santiago de Compostela, Spain; francisco.otero@usc.es; 4 Ophthalmology Department, University Clinical Hospital of Santiago de Compostela (SERGAS), 15706 Santiago de Compostela, Spain; maria.jose.rodriguez.cid@sergas.es (M.J.R.-C.); maria.gil.martinez@sergas.es (M.G.-M.); maximino.jose.abraldes@sergas.es (M.J.A.); 5 Instituto Oftalmológico Gómez-Ulla, 15706 Santiago de Compostela, Spain; franciso.gomez.ulla@sergas.es; 6 Department of Surgery, University of Santiago de Compostela, 15782 Santiago de Compostela, Spain; 7 FIDIS-Unidad de Epidemiología e Investigación Clínica, 15706 Santiago de Compostela, Spain; ana.estany.gestal@sergas.es

**Keywords:** ranibizumab, inflammatory, statins, uric acid, age-related macular degeneration

## Abstract

Anti-vascular endothelial growth factor drugs keep being the main therapy for neovascular age-related macular degeneration (AMD). Possible predictive parameters (demographic, biochemical and/or inflammatory) could anticipate short-term treatment response with ranibizumab. 46 treatment-naive patients were included in a prospective observational study. They underwent three monthly injections of intravitreal ranibizumab for neovascular AMD and the clinical examination was made at baseline and one month after the third injection. Demographic characteristics, co-morbidities and concomitant treatments were recorded at the baseline visit. Biochemical parameters, complete blood count and inflammation biomarkers were also measured at these times. Uric Acid was found to be statistically significant with a one-point difference between good and poor responders in both basal and treated patients, but only in basal parameters was statistical significance reached (*p* = 0.007 vs. *p* = 0.071 in treated patients). Cholesterol and inflammatory parameters such as white blood cell count and neutrophils were significantly reduced over time when treated with intravitreal ranibizumab. On the other hand, women seemed to have a worse prognosis for short-term response to intravitreal ranibizumab treatment. Uric acid may help identify possible non-responders before initial treatment with ranibizumab, and cholesterol and white blood cells could be good candidates to monitor short-term response to ranibizumab treatment.

## 1. Introduction

Age-related macular degeneration (AMD) is the leading cause of irreversible visual impairment among people older than 65 years worldwide [1]. Early stages of the disease are characterized by the presence and size of drusen and retinal pigment epithelium (RPE) alterations. The neovascular form (nAMD), an advanced form, is characterized by a severe vision loss caused by the growth of neo vessels under or within the macula [2].

AMD is considered a multifactorial disease where multiple factors such as genetic, and non-genetic factors intervene. Since the prevalence of the disease increases progressively with advancing age, this seems to be the most important risk factor [3,4]. Mutations in genes such as the *HTRA1/ARMS2*, complement factor H (*CFH*) and complement C3 have strong association with AMD disease [5]. On the other hand, several environmental risk factors, such as obesity [6], hypertension [7], smoking and hypercholesterolemia also seem to predispose to the development of AMD [8,9].

The pathophysiology of AMD is unclear and current evidence indicates that systemic and ocular inflammation also participates in the pathophysiology of nAMD [10], especially in retinal and choroid tissue. As a result of local stress, the body may respond with the recruitment of proinflammatory mediators and cytokines such as TNF, IL6 or IL8 to the affected tissue [11]. These inflammatory factors may also interfere with the balance of anti-angiogenic factors and promote choroidal neovascularization (CNV). Also, multiple genes related to complement cascade (*CHF*, *C3*) and inflammatory parameters (*IL8*) have been suggested to be related to AMD development [12]. Although various of these proinflammatory and proangiogenic mediators have been reported in the literature to be involved in nAMD, vascular endothelial growth factor (VEGF) keeps being the main target of nAMD therapy [13,14], while the treatment of geographic atrophy (GA) focuses on the complement cascade [15]. In any case, both treatments seek to stop the progression of the disease.

Ranibizumab is a recombinant fragment of a monoclonal antibody with a high affinity for VEGF-A. It was approved for the treatment of nAMD in 2006 following the phase III clinical trials MARINA and ANCHOR [16,17,18,19]. Most patients experience a significant reduction in symptoms after the intravitreal treatment but, regardless of the intervention a pool of patients end up showing a poor or no response to treatment [20]. Therefore, outcomes may be also affected by other individual factors such as comorbidities, concomitant treatments or treatment preferences. Different systemic drugs may interfere in the pathogenesis of AMD such as lipid-lowering drugs, non-steroidal anti-inflammatory drugs (NSAIDS) and antidiabetic drugs [21]. These drugs, in addition to their main effect for which they have been prescribed, they also have other systemic effects such as the reduction of inflammation and oxidative stress. Both pathways that are also part of the pathophysiology of AMD, so they could be used to prevent AMD or slow down progression, but not clear benefit was found in the long term [22].

Little is known about the effect these drugs may have on anti-VEGF treatment, especially when they are drugs commonly prescribed in AMD patients as they are usually elderly patients and these drugs are common concomitant treatments for their comorbidities [23]. Given that the high prevalence of AMD results in high socioeconomic burden associated with the anti-VEGF therapy, it is necessary to be able to predict treatment outcomes [24]. Looking for biomarkers can help identify new targets for treatment assessment. Therefore, the early identification of poor and non-responders is the key element for the implementation of individualized therapy in nAMD.

The aim of this study was to find possible predictive parameters (demographic, biochemical and/or inflammatory) that could be anticipate short-term treatment response with ranibizumab for nAMD treatment.

## 2. Materials and Methods

### 2.1. Characteristics of the Study Group

A prospective observational study of patients with untreated nAMD who were eligible for initiation of ranibizumab based on clinical practice. Patients were recruited and examined by ophthalmologists at the University Hospital of Santiago de Compostela. After an initial clinical examination (baseline measurements), patients received a loading dose with three monthly injections of intravitreal ranibizumab (Lucentis^©^, Novartis, Basel, Switzerland) and then followed a treat and extend regimen. Patients were evaluated using ophthalmoscopy, fluorescein angiography, optical coherence tomography (OCT), and OCT angiography. Research and data collection strictly adhered to the Declaration of Principles of Helsinki. Ethical approval was granted by the local institutional review board and the Autonomous Community of Galicia (2017/614).

### 2.2. Patient’s Criteria

Patients elected to be in this study had to be naïve to anti-VEGF therapy for nAMD, they had to give their consent to participate in this study and also for the collection and storage of blood samples. Patients had to meet the inclusion criteria that were defined as neovascular AMD, 55 years or older at diagnostic and they had to have a VA of more than 20 Early Treatment Diabetic Retinopathy Study (EDTRS) letters at baseline. Exclusion criteria included any retinal disease other than nAMD. In addition, patients who had undergone ophthalmic surgery other than cataract surgery or had signs of ocular inflammation were also excluded.

### 2.3. Treatment Response Analysis

Short-term response analysis to ranibizumab therapy was performed at the fourth month after the initiation of ranibizumab treatment. Patients were considered good responders when intraretinal fluid (IRF) and/or -subretinal fluid (SRF) was found to be resolved, central retinal thickening (CRT) was reduced over time and improvement of at least 5 ETDRS letters was found. Patients were considered poor or non-responders when decrease from baseline on OCT-CRT was less than 25%, no change in visual acuity (VA, <5 letters) was found and continued or new IRF, or SRF after anti-VEGF therapy appeared. This classification of treatment response was based on Amoaku et al. [25].

### 2.4. Data Collection

Demographic characteristics, body mass index (BMI), smoking history, co-morbidities and concomitant treatments were recorded at the baseline visit. Blood tests were performed at the baseline visit and one month after the loading phase, including basic biochemical parameters, complete blood count and inflammation biomarkers. For the biomarker measurement, plasma samples from the patients were separated from venous blood and stored frozen at −80 °C until analysis. The following cytokines were determined: IL1b, IL2R, IL5, IL6, IL8, IL10 and TNF-alpha using INMULITE analysers (Siemens Healthcare GmbH, Erlangen, Germany). INMULITE 1000 for IL2-R, IL-8, IL10 and TNF; and INMULITE 2000 for IL-6 and IL-2R. Reagents and instructions provided by the manufacturer were used in both devices.

### 2.5. Statistical Analysis

Data were analysed with SPSS Statistics 20^®^ (IBM SPSS Statistics) for Windows^®^. Wilcoxon signed-rank test was used to detect differences between parameter levels over time. Differences between good responder versus poor responder groups were explored using statistical tests; non-parametric Mann–Whitney test in the case of quantitative variables, and chi-square test or Fisher’s exact test in the case of categorical variables. A logistic regression analysis was performed to detect the association between demographic parameters, inflammatory parameters and response to ranibizumab treatment.

## 3. Results

### 3.1. Cohort Description

**Figure 1:**
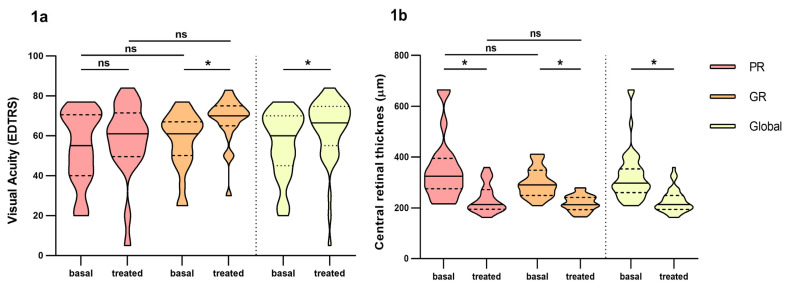
Differences in visual acuity and central retinal thickness between poor responders and good responders before and at fourth month of intravitreal ranibizumab treatment. (**a**): Visual Acuity (VA). (**b**): central retinal thickness. PR: Poor responder; GR: good responder. Wilcoxon signed-rank test was used to detect differences between parameter levels over time Mann–Whitney test was used to detect differences between groups. ns: *p*-value > 0.05, * <0.05.

Prospective observational study of 44 naïve patients. The demographic parameters of the study population are summarized in Table 1. Our sample study is composed of elderly people where 75% of the patients were over 75 years old at diagnostic with a median age of 80 years. We found 24 good responders vs. 20 poor responders in our group study. Visual acuity and central retinal thickness greatly improved over time between basal and treated patients, although we did not find differences between good responders a poor responders (Figure 1a,b). During study treatment and follow up, no ocular nor systemic adverse effects were reported from any of the patients.

**Figure 2:**
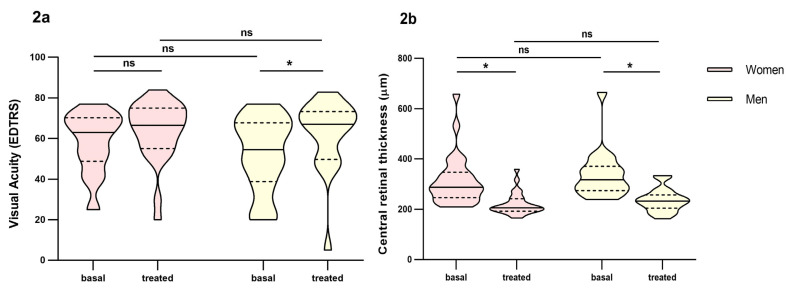
Differences in visual acuity and central retinal thickness between men and women before and at fourth month of intravitreal ranibizumab treatment. (**a**): Visual Acuity (VA). (**b**): central retinal thickness. Wilcoxon signed-rank test was used to detect differences between parameter levels over time Mann–Whitney test was used to detect differences between groups. ns: *p*-value > 0.05; * <0.05.

Women (59% of the patients) had worst outcomes than men in the short-term response to ranibizumab treatment (*p* = 0.014). Although in the overall count, women improved visual acuity, it did not reach statistical significance compared to men (Figure 2a). Also, women had more persistence of intraretinal, subretinal and subretinal pigment epithelium fluid (Table S1, Supplementary Material). Statin treatment seemed to favour a poor response to ranibizumab treatment in the short-term response (*p* = 0.068), although statistical significance was not reached.

### 3.2. Biochemical and Inflammatory Parameter Outcomes

**Figure 3:**
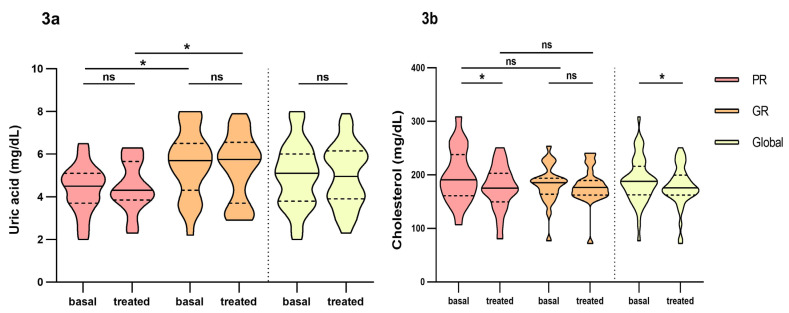
Differences in biochemical parameters uric acid and cholesterol between poor responders and good responders before and at fourth month of intravitreal ranibizumab treatment. (**a**): Uric Acid. (**b**): Cholesterol. PR: poor responder; GR: good responder. Wilcoxon signed-rank test was used to detect differences between parameter levels over time Mann–Whitney test was used to detect differences between groups. ns: *p*-value > 0.05; * <0.05.

**Figure 4:**
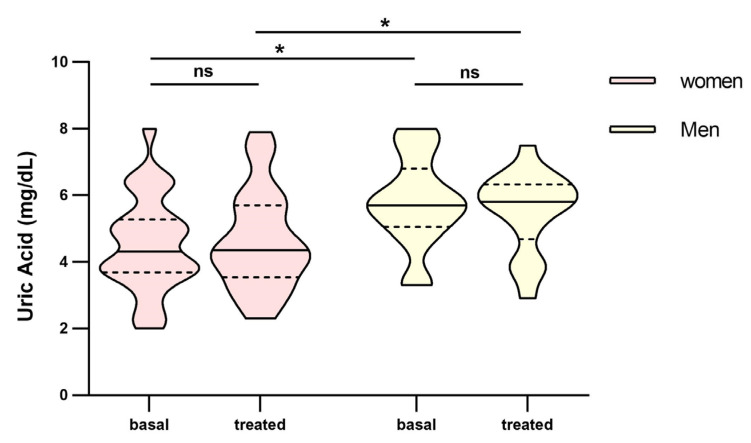
Differences in uric acid between men and women before and at fourth month of intravitreal ranibizumab treatment. Wilcoxon signed-rank test was used to detect differences between parameter levels over time Mann–Whitney test was used to detect differences between groups. ns: *p*-value > 0.05; * <0.05.

In the analysis between groups, we found that Uric Acid (UA) was found to be statistically significant between poor and good responders (Figure 3a). Difference over 1 point between groups in both basal (4.5 mg/dL IQR: 3.7–5.1 vs. 5.7 mg/dL, IQR: 4.3–6.5, respectively) and treated patients (4.4 mg/dL, IQR: 3.9–5.7 vs. 5.8 mg/dL, IQR: 3.7–6.6, respectively), but only in basal parameters did reach statistical significance (*p* = 0.007 vs. *p* = 0.071 in treated patients). Also, these parameters remained constant over time, both for poor and good responders (Figure 3a). Women had lower values of uric acid than men and it maintained over time (Figure 4).

Anyhow, these values are within the established therapeutic range (3.5–7.2 mg/dL).

Cholesterol was reduced over time in both good (186 mg/dL, IQR: 164–194 vs. 176 mg/dL, IQR: 162–190, *p* = 0.070) and poor responders (191 mg/dL, IQR: 161–238 vs. 175, mg/dL, IQR: 150–203, *p* = 0.015), but only did reach significance in the global count (188 mg/dL, IQR: 163–216 vs. 176 mg/dL, IQR: 162–200, *p* = 0.022) and poor responders (Figure 3b). No differences were found between groups and no other biochemical parameters were found significant.

**Figure 5:**
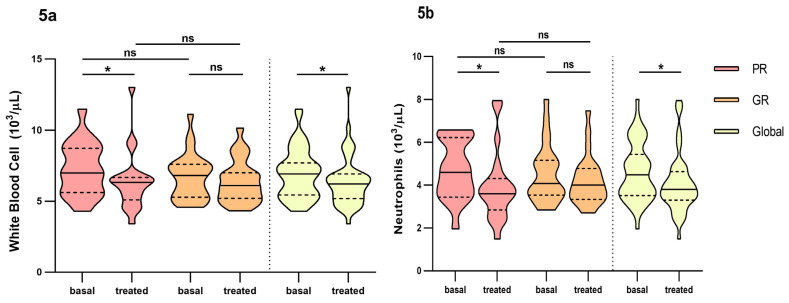
Differences in inflammatory parameters white blood cell and neutrophils between non-responders and good responders before and at fourth month of intravitreal ranibizumab treatment. (**a**): White blood cell. (**b**): Neutrophils. PR: Poor responder; GR: good responder, ns: *p*-value > 0.05; * <0.05. Wilcoxon signed-rank test was used to detect differences between parameter levels over time Mann–Whitney test was used to detect differences between groups. ns: *p*-value > 0.05; * <0.05.

We also found differences over time in global count for white blood cell count (WBC) (6.92 × 10^3/^, IQR: 5.44–7.70 vs. 6.22 × 10^3^, IQR: 5.20–6.93, *p* = 0.028) and poor responders (6.99 × 10^3^, IQR: 5.62–8.72 vs. 6.32 × 10^3^, IQR: 5.09–6.68, *p* = 0.030) (Figure 5a). The same for neutrophils (4.48 × 10^3^, IQR: 3.49–5.44 vs. 3.73 × 10^3^, IQR: 3.30–4.63, *p* = 0.012 in global count and 4.60 × 10^3^, IQR: 3.44–6.22 vs. 3.60 × 10^3^, IQR: 2.85–4.31, *p* = 0.044 in poor responders) (Figure 5b). We did not find any differences between groups in the short-term analysis of these parameter nor in the rest of the inflammatory parameters studied (Table S3, Supplementary Material). Of the cytokines analysed, IL6 and TNF alpha decreased over time and were over the therapeutic range while IL8 increased over time. None of them reached significance. IL1b, IL5 and IL10 remained unchanged over time (Supplementary Material).

A multivariate logistic regression analysis was performed with the variables that showed significance when it came to response in the univariate analysis. The univariate analysis was made with the parameters that were found significant in the short-term response analysis (Table 2). The parameters selected were sex, age, body mass index, smoker status, statins use, uric acid and creatinine. Patients under treatment with statins and women are prognostic factors for worse response to treatment with ranibizumab in the short-term response to ranibizumab treatment.

## 4. Discussion

Age is the main risk factor for AMD, and women seem to have a higher prevalence than men in elderly patients in Europe [26]. Long-term follow-up studies (14–15 years) show a higher incidence of lesions appearing in women in older age groups (>75 years) than men [3,4]. We found that women have a worse prognosis for treatment response. Also, 75% of our patients had a median age of 80 years old at diagnosis and beginning of treatment. The background for the poor prognosis found in females may be associated with the late start of treatment due to a late diagnosis compared to men. A study of Danish population [27] found that women, in the majority of the diseases, are diagnosed at an older age than men. We found no other studies with similar results in short-term response to treatment, but we found one study where males seemed to have a reduced effect of the anti-VEGF therapy on CRT but in a long-term analysis [28]. In other studies, sex was not found to predict outcomes [29,30].

On the other hand, elderly patients are usually treated with several drugs, including nonsteroidal anti-inflammatory drugs (NSAIDs), antihypertensives, antidiabetic and/or lipid-lowering agents which could interfere with pathways that also play a role in AMD pathogenesis and hence may affect it. Lipid-lowering drugs and antidiabetic drugs were associated with lower prevalent of any AMD type in Europe [21]. This study did not differentiate between statins or fibrates and metformin or insulin but its results explore the possible association of these drugs with AMD prevalence. However, little is known about the effect of concomitant treatments and the possible effect they may have in the treatment response of these patients to anti-VEGF injections for AMD. One study by Montero et al. found that concomitant systemic beta-adrenergic blocking agents may have a role in reducing the need for repeated bevacizumab intravitreal injections [31], but no other studies were found that investigated these possible associations.

Statins are known for lowering systemic cholesterol and reducing cardiovascular risk. But statins also have diverse effects on other pathophysiological pathways such as angiogenesis or inflammation [32]. Several studies promote the potential of this medication for the prevention and treatment of AMD [21,33], although others found no causal relationship [22]. We found no studies that related the effect of concomitant statins to AMD treatment. In our case, being on lipid-lowering treatment was associated with poor treatment outcomes to ranibizumab. Although unexpected, these results could be explained by the pro-vascular mechanism of statins that involves VEGF stimulation. In a mouse model that simulated clinical doses when administered simvastatin, they found that low levels of statins promoted vascular repair, but high doses increased the pathological neovascularisation when compared to controls [34].

On the other hand, we found that the biochemical parameter cholesterol was reduced over time, being this reduction bigger in the poor responders group, probably due to having a bigger population on statin treatment. Despite non-responders being on more statins, they have a similar basal cholesterol value between both groups and the lowering of cholesterol occurs in both groups, so it could be a good candidate to monitor response as this reduction was also observed other studies, although it did not reach significance [35,36]. These studies also measured lipoproteins such as HDL and LDL. Conflicting results have been reported with HDL concentration. High concentrations of this lipoprotein may increase the risk for any AMD form [37] and in consequence HDL dysfunction might also be implicated in AMD pathogenesis. But, these studies did not find any relation with anti-VEGF treatment. We did not measure lipoproteins separately but it would be interesting to know the lipid profile of our patients and find the possible associations of these lipoproteins to anti-VEGF treatment.

Uric acid (UA) is the end product of purine metabolism that has an anti-oxidative effect in normal circumstances. Treatment with UA was found to decrease retinal inflammation and vascular leakage in diabetic rats [38], but hyperuricemia led to structural changes [39,40,41]. Subramani et al. reported higher serum uric acid levels in nAMD patients compared to the control group, although it did not reach significance [39]. Horwath-Winter et al. found in cataract patients similar amounts of uric acid in tear fluid and aqueous humour, but they were significantly lower than those found in serum and these values were reduced in the course of the disease [42]. They also found that female cataract patients had lower uric acid values in tear fluid and aqueous humour compared with male cataract patients, this could also be related to circulating serum levels of uric acid where women have lower levels than men probably caused by hormonal regulation [43]. In our case, UA was found to be statistically significant between poor and good responders in this short-term response analysis with over 1 point of difference in the good-responder group. Also, women also had a one-point difference compared to men (Figure 4) and they were mostly in the poor-responder group. Anyhow, although this difference is not clinically relevant because both are in the established therapeutic range (3,5–7,2 mg/dL), it may help identify possible non-responders before initial treatment with ranibizumab as uric acid acts as an antioxidant and may have a protective effect and help to obtain a better response. So, this analytical parameter is an interesting parameter for future studies not only with a bigger population to corroborate our findings but also the possibility of obtaining aqueous fluid, or faster and more simple tear fluid measures of this parameter to determine the possible association between the antioxidative properties of uric acid and AMD and how it affects the treatment response.

The primary limitation of this study is the sample size. However, this limitation originates from the strict inclusion and exclusion criteria, along with the utilization of a sole centre for recruitment. Also, we analysed only short-term response as it allows for an improved understanding of the visual treatment potential and guidance on clinical management [25].

Finally, an overall reduction of systemic inflammation parameters was identified in this study including proinflammatory cytokines TNF-alpha and IL6, but these parameters did not reach statistical significance, similar to what other studies found [44,45]. We only found significance in WBC and neutrophils when the patients were treated with ranibizumab. We did not find similar results with other studies that used ranibizumab as anti-VEGF treatment [45]. Erdem et al. found significance in the reduction of these parameters when intravitreal aflibercept was used, but not when intravitreal ranibizumab was administered [36]. Moreover, we also found that poor responders had a significant change over time compared with good responders, but, since all fluctuations of these parameters remained within the stablished therapeutic range after the loading phase, it does not seem likely that these differences would have any clinical effect.

We did not find any differences in the other biochemical or inflammatory parameters, probably because ranibizumab is a monoclonal antibody fragment that does not have the FcRn (the neonatal Fc receptor for IgG). This FcRn protects it from lysosomal degradation [46], therefore, ranibizumab is rapidly eliminated from the systemic circulation with an estimated half-life 2 h [47]. This short-lived time may explain few effects on the systemic inflammatory parameters analysed.

## 5. Conclusions

Women seem to have had a worse prognosis to short-term treatment response to intravitreal ranibizumab. Patients on statin treatment also had a worse response to treatment. Uric acid had a one-point difference between good and poor responders. This difference may help identify possible non-responders before initial treatment with ranibizumab. Cholesterol and white blood cells could be a good candidate to monitor short-term response. Finally, the effect of short-term treatment with ranibizumab on biochemical and inflammatory parameters is uncertain. Nevertheless, further studies with larger sample size are needed to establish causality and to confirm the results found in this study.

## Figures and Tables

**Table 1:** Demographic parameters of study population.

Variables	Count N (%) (Total N = 44)	Poor Responders (N = 20)	Good Responders (N = 24)	*p*-Value *
Women	26, 59.1	16, 80.0	10, 41.7	0.014
Age; Median, IQR	80, (74–86)	80, (74–86)	79, (75–86)	0.850
Co-morbidities				
Obesity (>30 kg/m^2^)	17, 38.6	6, 30.0	11, 45.8	0.302
Smoker	14, 31.8	7, 35.0	7, 29.2	0.752
Smoking status Non-smoker smoker Ex-smoker	30, 68.2 3, 6.8 11, 25.0	13, 65.0 3, 15.0 4, 20.0	17, 70.8 0, 0.0 7, 29.2	0.134
Cardiovascular	30, 68.2	14, 70.0	16, 66.7	1.000
Endocrine	24, 54.5	14, 70.0	10, 41.7	0.076
Concomitant medication				
Anti-inflammatories	13, 29.5	7, 35.0	6, 25.0	0.522
Anticoagulants	9, 20.5	3, 15.0	6, 25.0	0.477
Statins	21, 47.7	13, 65.0	8, 33.0	0.068

* Mann–Whitney test was used to detect differences between groups.

**Table 2:** Univariate and multivariate analysis in short-term response to ranibizumab treatment with the demographic, biochemical parameters selected.

		Univariate Analysis		Multivariate Analysis	
Variable	N	OR (IC95%)	*p*-Value	OR (IC95%)	*p*-Value
Age	44	1.051 (0.971–1.137)	0.218		
women	44	0.179 (0.046–0.698)	0.013	0.153, (0.026–0.901)	0.038
Body Mass Index	44	1.039 (0.901–1.176)	0.669		
Smoker	44	0.765 (0.214–2.730)	0.679		
Statins	44	0.269 (0.077–0.940)	0.040	0.159, (0.032–0.794)	0.025
Uric Acid basal	43	1.894, (1.142–3.142)	0.013	1.595, (0.906–2.807)	0.105
Creatinine basal	43	42.387, (1.543–1164.734)	0.027		

## Data Availability

The data can be shared up on request.

## Supplementary Materials

The following supporting information can be downloaded at https://www.mdpi.com/article/10.3390/pharmaceutics16010105/s1. Table S1. Clinical parameters analyzed by sex; Table S2. Biochemical parameters analyzed by treatment response, Table S3. Inflammatory parameters results analyzed by treatment response.
