# Supplementary material for: The Effect of Systemic Parameters and Baseline Characteristics in Short-Term Response Analysis with Intravitreal Ranibizumab in Treatment-Naive Patients with Neovascular Age-Related Macular Degeneration
Source: Pharmaceutics. 2024 Jan 13;16(1):105. doi: 10.3390/pharmaceutics16010105 (PMC10818382; doi:10.3390/pharmaceutics16010105)
Supplement: Supplementary file 1 [file pharmaceutics-16-00105-s001.zip › pharmaceutics-2771479-supplementary.pdf]

Supplementary material: The effect of systemic parameters and baseline characteristics in short-term response analysis with intravitreal ranibizumab in treatment-naive patients with neovascular age related macular degeneration

Laura García-Quintanilla[1,2,3†], Pablo Almuiña-Varela[2,4†], María José Rodríguez-Cid[4], María Gil-Martinez[4], Maximino J Abraldes[4,5,6], Francisco Gomez-Ulla[5], Miguel González-Barcia[1,2], Cristina Mondelo-Garcia[1,2], Ana Estany-Gestal[7], Francisco J Otero-Espinar[3], Maribel Fernández-Rodríguez[4,5,6*], Anxo Fernández-Ferreiro[1,2*]

Table S1. Clinical parameters analysed by sex

	Total (n=44)	Women (n= 26)	Men (n= 18)	p-value
**Response parameters**				
AV (ETDRS)				
Basal	60, (45-70)	63, (49-70)	54, (39-68)	0.303
Treated	66, (55-75)	66, (55-75)	67, (50-73)	0.807
wilcoxon		0.057	0.003	
CRT (μm)				
Basal	298, (260-354)	288, (246-347)	318, (274-370)	0.214
Treated	214, (194-250)	206, (193-242)	232, (204-256)	0.170
wilcoxon	0.001	0.001	0.001	
IRF (N,%)				
Basal	27, 61.4	16, 61.5	11, 61.1	0.977
Treated	8, 18.1	6, 23.1	2, 11.1	0.425
SRF (N,%)				
Basal	33, 75.0	20, 76.9	13, 72.3	0.894
Treated	9, 20.5	7, 26.9	2, 11.1	0.270
SubRPE (N,%)				
Basal	30, 48.2	22, 84.6	8, 44.4	0.018
Treated	13, 29.5	11, 42.3	2, 11.1	0.043
**Biochemical parameters**				
Uric acid (mg/dL)				
Basal	5.1, (3.8-6.0)	4.3, (3.7-5.3)	5.7, (5.0-6.8)	0.008
Treated	4.9, (3.9-6.2)	4.4, (3.5-5.7)	5.8, (4.7-6.3)	0.031
wilcoxon	0.001	0.102	0.456	

Parameters: Median, IQR: interquartile range. CRT: central retinal thickness, ETDRS: Early Treatment Diabetic Retinopathy Study, IRF: Intraretinal fluid, SRF: subretinal fluid, RPE: retinal pigment epithelium, VA: Visual Acuity

Table S2. Biochemical parameters analysed by treatment response

	Total (N=44)	Poor responders N= 20	Good responders N= 24	p-value
**Urea (mg/dL)**				
Basal	44, (35-54)	42, (30-52)	46, (35-62)	0.212
Treated	43, (35-59)	43, (32-56)	46, (38-63)	0.339
Wilcoxon	0.567	0.749	0.558	
**Uric Acid (mg/dL)**				
Basal	5.1, (3.8-6.0)	4.5, (3.7-5.1)	5.7, (4.3-6.5)	0.007
Treated	4.9, (3.9-6.2)	4.4, (3.9-5.7)	5.8, (3.7-6.6)	0.071
Wilcoxon	0.516	0.618	0.254	
**Creatinine (mg/dL)**				
Basal	0.91, (0.72-1.07)	0.79, (0.66-0.97)	0.94, (0.82-1.16)	0.014
Treated	0.96, (0.72-1.08)	0.88, (0.68-1.01)	0.96, (0.82-1.14)	0.161
Wilcoxon	0.321	0.286	0.685	
**Cholesterol**				
Basal	188, (163-216)	191, (161-238)	186, (164-194)	0.385
Treated	176, (162-200)	175, (150-203)	176, (162-190)	0.981
Wilcoxon	0.022	0.015	0.070	
**Triglycerides**				
Basal	105, (71-145)	110, (71-154)	93, (70-116)	0.471
Treated	93, (55-146)	109, (54-157)	93, (69-132)	0.479
Wilcoxon		0.687	0.484	

Parameters: median, IQR. Reference range: Urea: 12-44 mg/dL; UA: 3,5-7,2 mg/dL, creatinine: 0,63-1,13 mg/dL;

Table S3. Inflammatory parameters results analysed by treatment response

	Total N=44	Poor responders N= 20	Good responders N= 24	p-value
**WBC ($x10^3$)**				
Basal	6.92, (5.44-7.70)	6.99, (5.62-8.72)	6.81, (5.28-7.60)	0.346
Treated	6.22, (5.20-6.93)	6.32, (5.09-6.68)	6.10, (5.20-7.01)	0.689
wilcoxon	0.028	0.030	0.290	
**Neutrophils ($x10^3$)**				
Basal	4.48, (3.49-5.44)	4.60, (3.44-6.22)	4.08, (3.54-5.16)	0.493
Treated	3.73, (3.30-4.63)	3.60, (2.85-4.31)	4.00, (3.33-4.78)	0.328
wilcoxon	0.012	0.044	0.141	
**CRP (mg/dL)**				
Basal	0.190, (0.120-0.410)	0.212, (0.120-0.610)	0.190, (0.115-0.392)	0.864
Treated	0.190, (0.111-0.470)	0.148, (0.087-0.556)	0.216, (0.122-0.395)	0.759
wilcoxon	0.599	0.904	0.411	
**ESR (mm)**				
Basal	22, (10-38)	22, (16-28)	21, (9-40)	0.525
Treated	22, (11-34)	20, (12-28)	25, (9-36)	0.663
wilcoxon	0.487	0.212	0.831	
**B2M (mg/L)**				

Basal<br>Treated	2.57, (2.00-3.35)<br>2.44, (2.10-3.48)	2.48, (1.98-3.20)<br>2.39, (2.02-3.28)	2.67, (1.99-3.69)<br>2.50, (2.25-3.62)	0.315<br>0.406
wilcoxon	0.407	0.201	0.954	
**TNF (pg/mL)**<br>Basal<br>Treated	 8.2, (7.0-10.8)<br>7.4, (6.5-10.4)	 7.8, (6.8-10.55)<br>7.2, (6.2-9.92)	 9.3, (6.7-12.1)<br>9.1, (6.9-10.5)	 0.232<br>0.154
wilcoxon	0.645	0.408	0.773	
**rIL-2 (U/mL)**<br>Basal<br>Treated	 466, (345-711)<br>476, (345-719)	 475, (317-610)<br>454, (328-660)	 461, (357-736)<br>505, (362-830)	 0.715<br>0.304
wilcoxon	0.301	0.877	0.184	
**IL-6 (pg/mL)**<br>Basal<br>Treated	 6.1, (4.2-8.2)<br>5.4, (3.3-7.8)	 6.1, (3.7-9.9)<br>5.2, (3.1-6.4)	 6.0, (4.3-8.2)<br>5.6, (3.8-8.2)	 0.893<br>0.215
wilcoxon	0.422	0.107	0.728	
**IL-8 (pg/mL)**<br>Basal<br>Treated	 22.0, (13.0-100.0)<br>33.0, (13.8-111.5)	 21.0, (13.0-100.0)<br>29.5, (12.2-81.0)	 24.0, (12.8-115.8)<br>37.0, (14.0-117.5)	 0.741<br>0.480
wilcoxon	0.102	0.546	0.092	

Parameters: median, IQR. B2M: Beta-2-Microglobulin, CRP: C-reactive protein, ESR: erythrocyte sedimentation rate, TNF: tumor necrosis factor; WBC: white blood count.
Reference range: B2M: 1,09-2,53 mg/L; CRP: 0-0,5 mg/dL: ESR: 0-20 mm; rIL-2: 158-623 U/mL; IL-6: 0-5 pg/mL; IL-8: 0-62 pg/mL; TNF: 0-8,1 pg/mL.
